# Dietary niches drive microbial community assembly, network reorganization, and symbiont evolution in freshwater fish gut microbiomes

**DOI:** 10.1093/ismejo/wrag125

**Published:** 2026-05-15

**Authors:** Hongye Shen, Jinhui Song, Jinshan Li, Yongmei Hu, Nan Peng, Shumiao Zhao

**Affiliations:** National Key Laboratory of Agricultural Microbiology and College of Life Science and Technology, Huazhong Agricultural University, Wuhan 430070, China; National Key Laboratory of Agricultural Microbiology and College of Life Science and Technology, Huazhong Agricultural University, Wuhan 430070, China; National Key Laboratory of Agricultural Microbiology and College of Life Science and Technology, Huazhong Agricultural University, Wuhan 430070, China; National Key Laboratory of Agricultural Microbiology and College of Life Science and Technology, Huazhong Agricultural University, Wuhan 430070, China; National Key Laboratory of Agricultural Microbiology and College of Life Science and Technology, Huazhong Agricultural University, Wuhan 430070, China; National Key Laboratory of Agricultural Microbiology and College of Life Science and Technology, Huazhong Agricultural University, Wuhan 430070, China

**Keywords:** freshwater fish, gut microbiota, metagenomic sequencing, potential probiotics, *Cetobacterium*

## Abstract

Host diet is a fundamental ecological factor shaping the assembly and evolution of host-associated microbiomes, yet how dietary niches influence the structure of microbial associations and functional adaptation in freshwater fish remains poorly understood. This study selected five dominant farmed freshwater fish species in China with distinct feeding habits (herbivory, omnivory, filter-feeding, and carnivory) and systematically investigated the adaptive mechanisms of their gut microbiomes by integrating metagenomics, targeted cultivation, comparative genomics, and *in vitro* assays. We show that dietary niches exert a strong deterministic effect on microbial community assembly, leading to pronounced differences in ecological network topology, including connectivity, modularity, and keystone taxa. *Cetobacterium* was detected in all five fish species but exhibited a higher relative abundance in omnivorous (16.0%) compared to carnivorous fish (5.4%), suggesting that it may be a core genus within the gut microbiota of freshwater fish. Comparative genomics further revealed that *Cetobacterium* symbionts exhibit streamlined genome architectures and conserved core metabolic functions, indicative of adaptive evolution toward stable host-associated lifestyles. Guided by metagenomic insights, we isolated multiple *Cetobacterium* strains displaying host-adapted functional traits, linking community-level ecological patterns to cultivable symbiont resources. In summary, our findings demonstrate that freshwater fish guts function as ecological niches that deterministically structure microbial community assembly and drive symbiont evolution, providing a conceptual framework for understanding host-microbiome co-adaptation in aquatic ecosystems.

## Introduction

The animal gut is a complex ecosystem in which the microbiome plays a central role in host nutrient metabolism, immune development, and disease resistance, functioning as the host’s “second genome” [1–3]. These microorganisms generate hydrolytic enzymes that ferment dietary carbohydrates into utilizable energy, whereas their metabolic products also regulate intestinal barrier integrity and modulate systemic physiology [4, 5]. For example, microbial metabolites activate G-protein-coupled receptors to modulate host physiology, and short-chain fatty acids (SCFAs), such as butyrate, exert anti-inflammatory effects and promote the maturation of regulatory T cells [6, 7].

Freshwater fish, owing to their naturally extensive dietary diversity, represent important model systems for investigating host-microbiome associations [8]. As the global leader in aquaculture, China contributes ~60.1% of worldwide production, with freshwater systems accounting for over 56.4% [9–12]. Freshwater aquaculture in China is predominantly based on cyprinid species, including *Ctenopharyngodon idella* and *Hypophthalmichthys molitrix*, and also includes high-value species exemplified by *Siniperca chuatsi*, *Micropterus salmoides*, and *Pelteobagrus fulvidraco* [9]. Their diverse feeding strategies render the fish gut a natural model for investigating diet-driven microbiome adaptation and functional evolution [1, 13]. Although taxonomic shifts and community assembly in these species are well-documented [14], much of this knowledge remains at the community-wide level. There remains a critical knowledge gap in understanding how gut symbionts of freshwater fish, such as *Cetobacterium*, utilize pan-genome plasticity and strain-level diversification to achieve functional specialization across such divergent dietary niches.

The fish gut microbiome is pivotal for host health, promoting nutrient absorption, growth performance, immune modulation, and pathogen suppression [8, 15]. And gut homeostasis supports health; dysbiosis can impair immune function and increase disease susceptibility [16]. Certain strains exert probiotic effects through unique physiological traits [17]. For instance, *Cetobacterium somerae*, a dominant taxon in fish guts, synthesizes vitamin B12 (VB_12_) to support host health and mediates ammonia detoxification [14, 18, 19]. However, a systematic understanding of gut microbial diversity among fish with different feeding habits, and of the effects of diet on gut microbiome composition, remains lacking. Despite their superior production volume, freshwater fish microbiomes have historically been overlooked; ~75% of aquaculture research between 2000 and 2020 focused on marine systems, leaving a critical knowledge gap in our understanding of freshwater gut ecosystems [12, 20].

Methodologies for studying the fish gut microbiome have evolved from 16S rRNA gene amplicon sequencing [21–23] to metagenomic reconstruction, which enables metagenome reconstruction, antibiotic resistance gene (ARG) profiling, and the exploration of potential probiotic resources [24, 25]. Although diet is a key driver of microbial composition [26], significant gaps remain in understanding ecological associations and functional validation [13, 27–29]. First, although the co-occurrence networks of fish-associated microbiomes for specific species under different environmental and rearing conditions have been characterized, there remains a limited number of studies that systematically compare the network topologies across different fish families and dietary groups [14, 28, 30, 31]. Second, microbial functions inferred from sequencing data lack validation at the level of cultivable strains. This limitation directly leads to a third issue: core symbiotic microorganisms have not been systematically evaluated in terms of phylogenetic diversity, cultivation characteristics, or probiotic potential, resulting in delayed strain resource development [19].

To address knowledge gaps, we conducted a multi-tiered investigation—from community ecology to probiotic development—using five economically important freshwater fish species with distinct feeding strategies (herbivorous, omnivorous, filter-feeding, and carnivorous). First, metagenomic analysis was employed to resolve shifts in taxonomic composition, functional potential, and co-occurrence associations across dietary groups, clarifying diet-driven assembly processes. Guided by these metagenomic findings, core symbionts with potential probiotic traits, specifically *Cetobacterium*, were isolated to establish a strain library. Finally, representative strains underwent polyphasic taxonomic characterization and whole-genome sequencing to assess organic acid production, *in vitro* probiotic functions, and safety profiles. By integrating ecological patterns with functional traits, this study systematically elucidates diet-driven gut microbiome assembly in freshwater fish, providing a scientific foundation for probiotic development and sustainable health management in aquaculture.

## Materials and methods

### Sample collection and strain isolation

Five important freshwater aquaculture fish species with distinct feeding habits were selected for this study: the herbivorous *C. idella*, filter-feeding *H. molitrix*, carnivorous *M. salmoides* and *S. chuatsi*, and the omnivorous *P. fulvidraco*. Samples were collected in September 2024 from a centralized aquaculture zone in Xianning, Hubei Province, China (30°01′N, 114°17′E). To minimize the confounding effects of geographical and environmental heterogeneity, all target fish species (*n* = 6 per species, totaling 30 individuals) were sampled from adjacent, hydrographically-linked ponds managed under a standardized “natural farming status” protocol. All fish appeared healthy and exhibited no clinical symptoms of disease at the time of collection.

For bacterial strain isolation, the intestinal sample homogenate was serially diluted 10-fold using sterile phosphate-buffered saline (PBS). Aliquots (100 μl) from dilution gradients of 10^−2^ to 10^−5^ were spread onto Brain Heart Infusion (BHI) agar plates, with three replicate plates prepared for each dilution [24, 32]. Plates were incubated anaerobically at 37°C for 48 h. Morphologically distinct colonies were picked and purified via successive streaking on fresh BHI agar to obtain axenic cultures.

### Deoxyribonucleic acid (DNA) extraction, library preparation, and sequencing

Total genomic DNA was extracted from the 30 fish gut content samples and all selected bacterial isolates using the MagBeads FastDNA Kit for Soil (MP Biomedicals, CA, USA), following the manufacturer’s instructions, and stored at −20°C prior to further assessment. Details of the sequencing throughput are provided in Supplementary material Appendix 1.

### Metagenomic data analysis methods

Raw sequencing reads were processed to obtain quality-filtered reads for further analysis. First, this involved adapter removal and trimming of low-quality reads using fastp (v 0.23.2) [33]. Subsequently, reads were aligned to the respective host genome using Minimap2 (version 2.24-r1122). Then, MEGAHIT (version 1.1.2) software was used to concatenate and assemble the valid read sequences of each sample, with contigs of no <300 bp length retained by default [34]. Prodigal (version 2.6) was used (https://github.com/hyattpd/Prodigal/) to identify open reading frames and predict the coding regions to obtain corresponding gene and protein sequence files. MMseqs2 (v13.45) was used to remove redundancy and obtain non-redundant gene and protein sets [35]. Taxonomic classification of each sample was executed using Kraken2 (v2.0.8-beta) with the “—confidence 0.5” option against a customized database [36].

For the obtained non-redundant protein set, species annotation was performed using mmseq2 (version 13–45 111). The database comprised nucleotide sequences from prokaryotic and eukaryotic microorganisms of Genome Taxonomy Database (GTDB) nt, as well as nucleotide sequences from viruses of Reference Viral Database (RVDB). Use the Strobealign (version 0.12.0) plugin to map the high-quality reads of each sample onto the overlapping group, and make the featureCounts (version 2.0.3). Count reads to determine gene abundance. Using mmseq2 in "search" mode with parameter “- s 5.7”, functional number annotations were performed on databases including Kyoto Encyclopedia of Genes and Genomes (KEGG), evolutionary genealogy of genes: Non-supervised Orthologous Groups (eggNOG), Gene Ontology (GO), Carbohydrate-Active Enzymes Database (CAZy), and Comprehensive Antibiotic Resistance Database (CARD) to obtain species abundance tables at various classification levels and abundance tables at various functional levels [37–39].

### Genome preparation, sequencing, and annotation

#### Preparation of bacterial biomass for sequencing

To obtain high-quality genomic DNA, bacterial strains were recovered from −80°C glycerol stocks and streaked onto Generalized Additive Model (GAM) agar plates, followed by anaerobic incubation at 37°C for 48 h. A single colony was inoculated into GAM broth and subcultured into fresh medium at 1.0% (v/v). Cells were harvested by centrifugation at 4000 × g for 10 min at 4°C, washed with prechilled sterile PBS, resuspended in a small volume of PBS, and pelleted again. Cell pellets were immediately flash-frozen in liquid nitrogen and stored at −80°C until DNA extraction [24].

#### Whole-genome sequencing, assembly, and annotation

Comparative genomic analysis was conducted using a hybrid sequencing and assembly strategy. Sequencing was performed on the NovaSeq 6000 system (Illumina) and Oxford Nanopore Technologies platforms. Flye [40] and Unicycler [41] software was used to assemble the data. Subsequently, all assembled results were integrated to generate a complete sequence. Finally, the genome sequence was acquired after the rectification by using pilon [42].

#### Measurement of antioxidant capacity

Due to the inter-strain variation, there are differences in the distribution of antioxidant substances. The fermentation supernatant and cell suspension of the selected and control strains were used to measure antioxidant capacity. Detailed experimental protocols and reagent specifications are provided in Supplementary material Appendix 2.

### *In vitro* safety assessment

#### Hemolytic activity

Hemolytic activity was evaluated by streaking *Cetobacterium* strains onto Columbia blood agar plates, with *Staphylococcus aureus* used as a positive control. Plates were incubated anaerobically at 37°C for 48 h. Hemolysis was assessed by observing the presence of clear zones (β-hemolysis), greenish zones (α-hemolysis), or the absence of hemolysis (γ-hemolysis) around the colonies. Detailed experimental protocols and reagent specifications are provided in Supplementary material Appendix 3.

#### Antibiotic susceptibility testing

Antibiotic susceptibility was determined using the Kirby-Bauer disk diffusion method in accordance with Clinical and Laboratory Standards Institute (CLSI) guidelines. Activated bacterial cultures were adjusted to an OD_600_ of 0.8 ± 0.05 and evenly spread onto GAM agar plates using sterile glass beads. After drying, antibiotic disks were placed onto the agar surface using sterile forceps. Plates were incubated anaerobically at 37°C for 48 h, and inhibition zone diameters were measured using a digital caliper. Results were interpreted as susceptible (S), intermediate (I), or resistant (R) according to CLSI criteria. All assays were performed in triplicate [24].

### Statistical analysis

Data manipulation and visualization were performed through the R package tidyverse (1.3.0) [43]. To account for varying sequencing depths, samples were rarefied to a uniform depth of 60 000 reads prior to analysis. Alpha diversity (Shannon, Pielou’s evenness, Observed species, and Faith’s PD) and beta diversity (Jaccard and Bray-Curtis dissimilarities) were calculated using the vegan package [44, 45]. Continuous variables were compared using *t*-tests, Kruskal–Wallis, or Wilcoxon rank-sum tests where appropriate. Phylogenies were inferred using IQ-TREE with automatic model selection [46]. Detailed computational pipelines and statistical parameters for the microbial co-occurrence network analysis are documented in Supplementary material Appendix 4. Comprehensive methodologies regarding the comparative genomics of *Cetobacterium* isolates, including pangenome modeling, collinearity analysis, and functional annotation parameters, are provided in Supplementary material Appendix 5. All visualizations were generated in R, mainly based on ggplot2.

## Results

### Significant differences in gut microbial community composition among five farmed freshwater fish species

**Figure 1:**
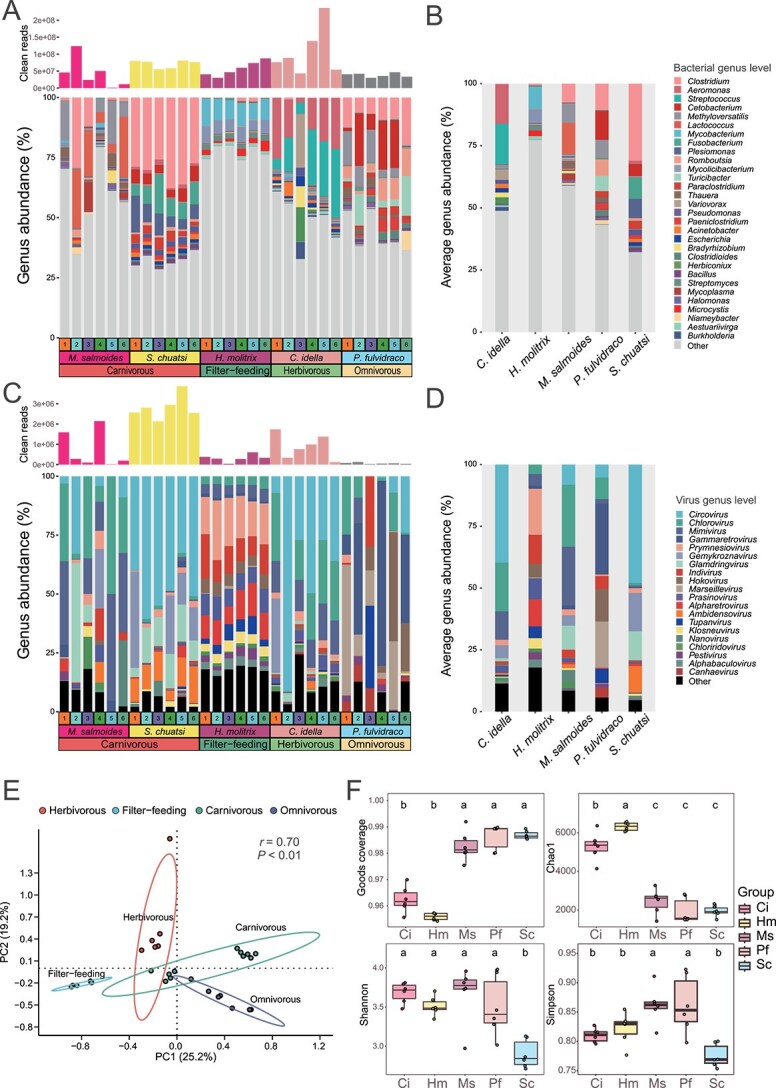
Gut microbiome profiles of five freshwater fish species with distinct feeding habits. (A) Genus-level bacterial community composition across individual samples. (B) Average relative abundance of dominant bacterial genera in each fish species. (C) Genus-level viral community composition across individual samples. (D) Average relative abundance of dominant viral genera in each fish species. (E) PCA based on species-level relative abundances. (F) Alpha diversity metrics, including Good’s coverage, Chao1 richness, Shannon diversity, and Simpson index. Different letters indicate significant differences among groups (*P* < .05).

Metagenomic sequencing was performed on five freshwater fish species: the carnivorous *M. salmoides* and *S. chuatsi*, the filter-feeding *H. molitrix*, the herbivorous *C. idella*, and the omnivorous but carnivory-biased *P. fulvidraco*. Dominant bacterial genera exhibited distinct distribution patterns across dietary groups (Fig. 1A and B). In carnivorous and carnivory-biased fish, the genus *Clostridium* (34.7%, phylum Firmicutes) predominated, typically accounting for more than 20.0% of the gut microbiota, consistent with protein and lipid-rich dietary requirements. In contrast, the gut microbiota of the herbivorous *C. idella* was significantly enriched in phylum Proteobacteria, particularly the genus *Aeromonas* (21.4%), whereas the filter-feeding *H. molitrix* showed enrichment of phylum Actinobacteria, dominated by *Mycobacterium* (14.8%) and *Mycolicibacterium* (9.3%). These findings show a consistent correspondence between feeding strategy and gut bacterial community composition. *Cetobacterium* was detected in the gut microbiomes of all five fish species and exhibited relatively high abundance in *P. fulvidraco* and *S. chuatsi* (Fig. 1A and B), identifying it as a ubiquitous core member of the freshwater fish gut microbiome.

Metagenomic data also revealed the presence of viral, eukaryotic, and archaeal sequences; however, their overall relative abundances were low (<5%, Fig. 1C and D). Viral communities were detected across all five fish species and were primarily composed of *Circovirus*, *Chlorovirus*, and *Mimivirus*, with the lowest viral abundance observed in *P. fulvidraco*. Eukaryotic microorganisms were mainly composed of fungi, algae, and a small proportion of protozoa (Fig. S1A and B), whereas archaeal communities were dominated by methanogenic and ammonia-oxidizing archaea (Fig. S1C and D). Overall, bacteria overwhelmingly dominated the gut across all five species.

Principal coordinates analysis (PCA) based on species-level relative abundances revealed significant separation of gut microbial community structures among fish with different feeding habits (*P* < .01, Fig. 1A). The filter-feeding *H. molitrix* clustered independently, whereas carnivorous species and the omnivorous *P. fulvidraco* grouped more closely. Analysis of Shannon and Simpson indices showed that *S. chuatsi* harbored significantly lower microbial diversity than other species, indicating a specialized gut microbiota.

### Co-occurrence network analysis reveals distinct microbial interaction patterns across fish with different feeding habits

**Figure 2:**
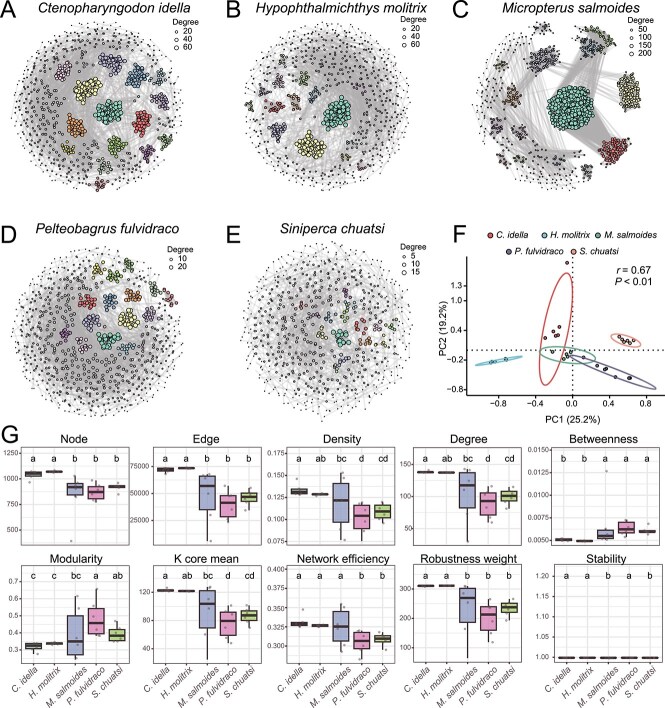
Gut microbial co-occurrence networks across freshwater fish species with different feeding habits. (A–E) Co-occurrence networks of gut microbiota constructed for five freshwater fish species: *C. idella* (A), *H. molitrix* (B), *M. salmoides* (C), *P. fulvidraco* (D), and *S. chuatsi* (E). Nodes represent microbial taxa; node size is proportional to degree. Colors indicate network module. (F) PCA of network topological features. (G) Comparison of network topology under different treatments. Different letters indicate significant differences among treatments at *P* < .05 (multiple comparisons with Kruskal–Wallis tests).

To evaluate community organization, we constructed gut microbial co-occurrence networks for five freshwater fish species (Fig. 2A–E). Topological analysis revealed that dietary niches significantly influence network architecture (Fig. 2G). The carnivorous species, particularly *M. salmoides*, exhibited high connectivity and complexity, characterized by elevated node degrees and network efficiency (Fig. 2G). In contrast, the gut microbial co-occurrence networks of the herbivorous *C. idella* and filter-feeding *H. molitrix* displayed higher modularity (Fig. 2G). These modular networks represent a more compartmentalized community structure, potentially reflecting specialized functional niches within the gut environment.

Statistical comparisons of network properties further highlighted these differences. *C. idella* and *H. molitrix* maintained higher stability and robustness compared to the carnivorous species (Fig. 2G). Specifically, metrics such as betweenness centrality and modularity were significantly higher in *P. fulvidraco* and *S. chuatsi* compared to the herbivorous and filter-feeding groups (*P* < .01, Fig. 2G). PCA confirmed clear differentiation among the co-occurrence structures (Fig. 2F), carnivorous species clustered along PC1 (25.2%), whereas *H. molitrix* and *C. idella* formed distinct groups, reflecting their unique organizational strategies (Fig. 2F).

### Gut microbial functional profiles exhibit high synergy with host dietary habits

**Figure 3:**
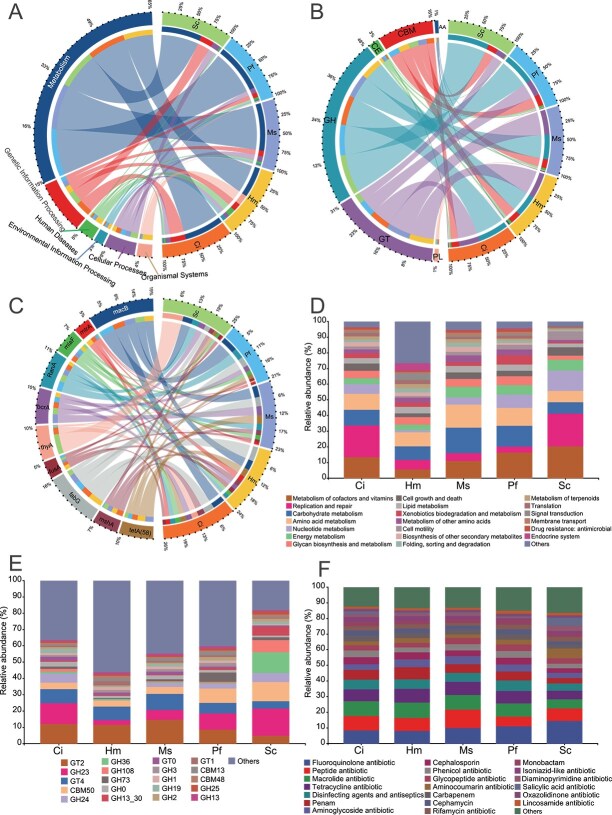
Functional profiling of gut microbiota across five freshwater fish species with distinct feeding habits, (A) KEGG level 1 functional annotation of the gut microbiota. (B) CAZyme gene families involved in carbohydrate metabolism, with the abundance of GHs and CEs in different fish species. (C) Summary of key ARGs enriched in fish species with distinct feeding habits. (D) KEGG level 2 functional annotation of microbial gene functions. (E) Gene-level annotation of ARGs across different fish species, highlighting differences in environmental antibiotic resistance profiles. (F) Summary of key ARGs (level 2 functional annotation) enriched in fish species with distinct feeding habits.

Gut metagenomes were annotated against the KEGG, CAZyme, and CARD databases to characterize functional potential. KEGG Level 1 annotation revealed that gut microbial communities across all fish species were predominantly involved in core metabolic and genetic information-processing pathways, including carbohydrate metabolism, amino acid metabolism, replication and repair, and translation (Fig. 3A). At higher resolutions, including KEGG Level 2 pathways, CAZyme family classifications, and ARG profiles, gut microbial functions exhibited distinct structural shifts associated with host trophic strategies (Fig. 3B, D, and  F). With respect to carbohydrate degradation, the gut microbiome of the herbivorous fish *C. idella* exhibited a specialized potential for plant polysaccharide decomposition, characterized by an expansion of genes encoding glycoside hydrolases (GHs) and carbohydrate esterases (CEs). Several GH families (e.g. *GH3*, *GH5*, and *GH43*) were notably prevalent, supporting plant cell wall components [47]. This CAZyme repertoire aligns with the plant-based diet of *C. idella*, reflecting the functional specialization of its gut microbiome for the utilization of plant-derived substrates.

Carnivorous fish species (*M. salmoides* and *S. chuatsi*) harbored lower abundances of carbohydrate metabolism, but exhibited enhanced potential for amino acid and energy metabolism (Fig. 3D). This aligns with a diet dominated by animal-derived substrates. Analysis of ARGs further revealed divergent selective pressures acting on gut microbiomes under different trophic strategies and ecological contexts (Fig. 3F). For example, *S. chuatsi* exhibited enrichment of the thyA gene associated with para-aminosalicylic acid tolerance, whereas *H. molitrix* showed higher abundance of the fabG (isoniazid resistance). These differential ARG distributions reflect microbial adaptation to host trophic niches and highlight the potential influence of antibiotic contamination in aquaculture environments on gut microbial assemblages.

### Functional enrichment analysis reveals differential enrichment of gut microbial functions across different hosts

**Figure 4:**
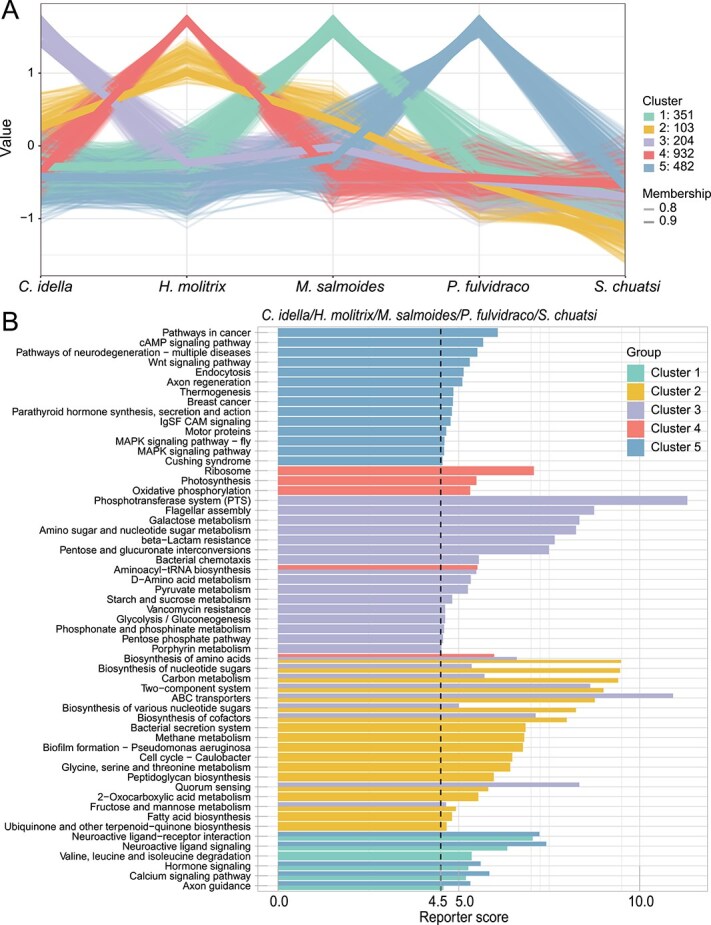
Functional enrichment analysis reveals host-dependent enrichment of gut microbiome functions. (A) Clustering analysis based on KEGG pathway reporter scores divides all functional pathways into five major clusters (cluster 1–5), with each cluster exhibiting specific enrichment patterns in different dietary groups. (B) The biological significance of the functional modules was further analyzed, showing that they are closely related to the host's nutritional strategy.

KEGG pathways were partitioned into five distinct clusters based on their Reporter Score profiles, reflecting divergent microbial functional investment (Fig. 4A). Hierarchical clustering based on KEGG pathway Reporter scores partitioned all functional pathways into five major clusters (Clusters 1–5), each representing a distinct functional guild aligned with host dietary niches (Fig. 4A). Cluster 3 was enriched in carbohydrate metabolism and glycan degradation pathways, showing a peak in herbivorous species. Cluster 1 and Cluster 5 primarily comprised environmental information processing and amino acid turnover, predominantly associated with carnivorous hosts. Cluster 2 and Cluster 4 encompassed core genetic processing and energy metabolism pathways, reflecting fundamental microbial maintenance across all species (Fig. 4B).

The *C. idella* gut microbiome was significantly enriched in Cluster 3, which included galactose metabolism, pentose and glucuronate interconversions, pyruvate metabolism, and starch and sucrose metabolism, as well as amino acid metabolism-related pathways such as amino sugar and nucleotide sugar metabolism and D-amino acid metabolism. In contrast, the filter-feeding *H. molitrix* uniquely exhibited significant enrichment in Clusters 2 and 4, comprising carbon and methane metabolism, alongside colonization-related pathways (two-component and bacterial secretion systems). The carnivorous fish *M. salmoides* showed significant enrichment in Cluster 1, featuring branched-chain amino acid degradation (valine, leucine, and isoleucine) and signaling processes (hormone and calcium signaling pathways).

The omnivorous *P. fulvidraco* gut microbiome exhibited pronounced enrichment in Cluster 5, encompassing multiple signal transduction pathways, including the cAMP, Wnt, and MAPK signaling pathways (Fig. S2). *Siniperca chuatsi* did not exhibit significant enrichment in the defined clusters, it exhibited higher relative abundances in secondary bile acid biosynthesis (map00121), protein digestion and absorption (map04974), and riboflavin metabolism (map00740). Overall, enrichment of gut microbial functions was strongly influenced by host trophic strategies, with distinct fish species exhibiting markedly different functional enrichment patterns.

### Large-scale isolation of *Cetobacterium* species and identification of new species guided by metagenomics

**Figure 5:**
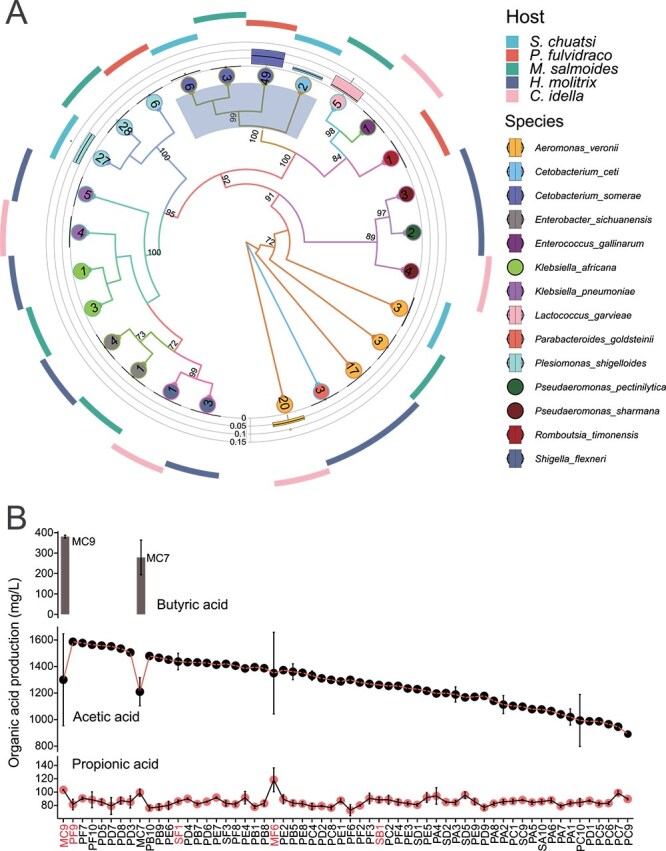
Genomic features and functional potential of *Cetobacterium* strains SF1 and other isolates. (A). Unrooted maximum-likelihood phylogenetic tree based on near-complete 16S rRNA gene sequences (Sanger sequencing) from diverse host species. Values at nodes represent bootstrap support from 1000 replicates calculated via IQ-TREE; only values >70 are shown for clarity. *Cetobacterium* from *M. salmoides* (MC9), *P. fulvidraco* (PF9), and *S. chuatsi* (SF1) are highlighted to show their evolutionary divergence. (B) Organic acid production profiles of different *Cetobacterium* strains. All strains showed acetic and propionic acid production, with *M. salmoides* (MC9) and *P. fulvidraco* (PF9) strains showing the highest levels of acetic acid.

Identification of *Cetobacterium* members as ubiquitous core symbiotes, we performed targeted anaerobic cultivation to obtain representative strains. A total of 236 pure bacterial isolates were obtained and preserved from the gut of the five fish species. Based on 16S rRNA gene sequencing and phylogenetic analysis (Fig. 5A), 60 strains were assigned to the genus *Cetobacterium*, including 49 strains isolated from *M. salmoides*, three from *P. fulvidraco*, and eight from *S. chuatsi*. Phylogenetic reconstruction revealed that the 60 *Cetobacterium* isolates (Fig. 5A) formed a well-supported monophyletic clade, with high bootstrap values indicating robust phylogenetic placement. Isolate SF1 shared only 98.32% 16S rRNA gene sequence identity with *Cetobacterium ceti*, falling below the commonly accepted threshold for bacterial species delineation. These results suggest that this isolate likely represents a new species within the genus *Cetobacterium*.

Representative *Cetobacterium* isolates were evaluated for SCFA production (Fig. 5B). All tested isolates produced acetate and propionate, with acetate concentrations ranging from 1200–1600 mg/l and propionate concentrations ranging from 80–125 mg/l. Strains PF9 and MF6 exhibited the highest yields. In contrast, butyrate production was strain-specific and was detected only in strains MC9 and MC7, both isolated from *M. salmoides*.

### Comparative genomics reveals species differentiation and genetic diversity of the genus *Cetobacterium*

**Figure 6:**
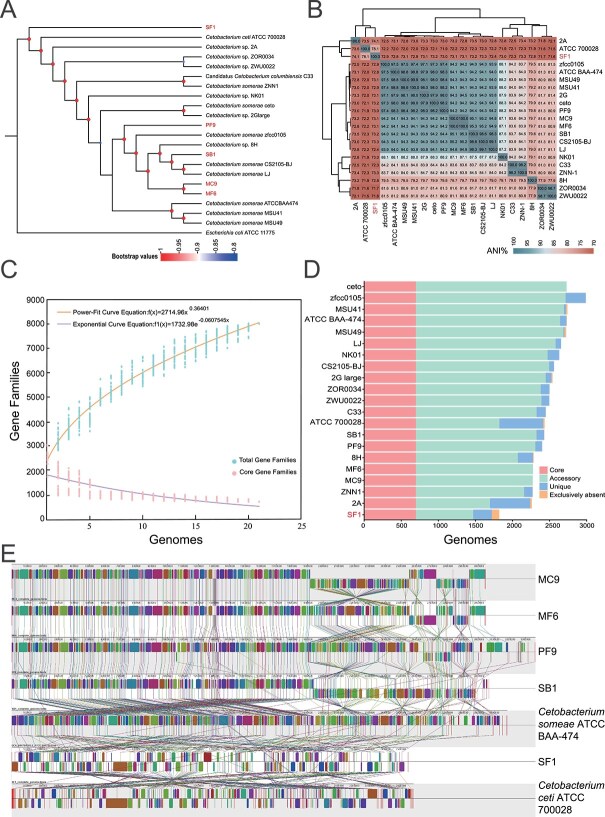
Comparative genomic analysis reveals species divergence and genomic diversity within the genus *Cetobacterium*. (A) Phylogenetic tree constructed based on core gene sequences of *Cetobacterium* genomes, with newly isolated strains highlighted. Bootstrap values are represented by node colors. (B) Heatmap of pairwise ANI values among *Cetobacterium* genomes, illustrating genomic relatedness and species boundaries. (C) Pan-genome and core-genome accumulation curves showing an open pan-genome structure of the genus. (D) Distribution of core, accessory, unique, and exclusively absent gene families across individual *Cetobacterium* genomes. (E) Co-occurrence network of core gene families, revealing a modular genomic organization and distinct clustering of the newly identified *Cetobacterium* SF1.

To resolve the phylogenetic relationships and population genetic structure of the genus *Cetobacterium*, five representative isolates were selected for whole-genome sequencing, including strains SB1 and SF1 isolated from *S. chuatsi*, strains MC9 and MF6 isolated from *M. salmoides*, and strain PF9 isolated from *P. fulvidraco*. These were analyzed alongside 20 publicly available genomes, totaling 25 strains (Table S1) [48]. Phylogenetic trees were independently constructed based on concatenated core gene sequences and whole-genome single-nucleotide polymorphisms (Fig. 6A). This consistency indicates that the evolutionary history of *Cetobacterium* is predominantly shaped by vertical inheritance, with minimal evidence of confounding horizontal gene transfer in core lineages.

The strain SF1 formed a well-supported sister clade to *C. ceti*, representing an evolutionary lineage distinct from *C. somerae*. To quantitatively validate this inference, average nucleotide identity (ANI) was calculated for all genome pairs. The ANI heatmap (Fig. 6B) was highly consistent with the phylogenetic tree, with SF1 exhibiting an ANI value of only 78.1% relative to its closest reference genome, *C. ceti* ATCC 700028, far below the widely accepted species delineation threshold (95%–96%). These genome-wide results unambiguously confirm SF1 as a new species within the genus *Cetobacterium*.

Pan-genome analysis revealed that the total gene repertoire expanded continuously as more genomes were incorporated, consistent with an open pan-genome model (f(x) = 2714.96x^0.30401^, Fig. 6C). In contrast, the core genome rapidly converged and stabilized at ~1700 gene families, indicating that *Cetobacterium* maintains highly conserved core functions that exhibiting substantial genetic plasticity, which likely enables adaptation to diverse ecological niches through the gain and loss of accessory genes.

Fine-scale comparison of gene content substantiated this conclusion (Fig. 6D). Although core genes predominated in all genomes, the proportions of accessory and strain-specific genes differed markedly among strains. *Cetobacterium* SF1 harbored a distinct set of strain-specific genes and exhibited partial loss of certain gene families, traits that potentially drive its functional divergence and ecological specialization. Consistently, the core gene family co-occurrence network displayed a pronounced modular organization (Fig. 6E), in which SF1 was clearly segregated from other *Cetobacterium* strains.

### Genomic features of representative *Cetobacterium* strains reveal probiotic potential

**Figure 7:**
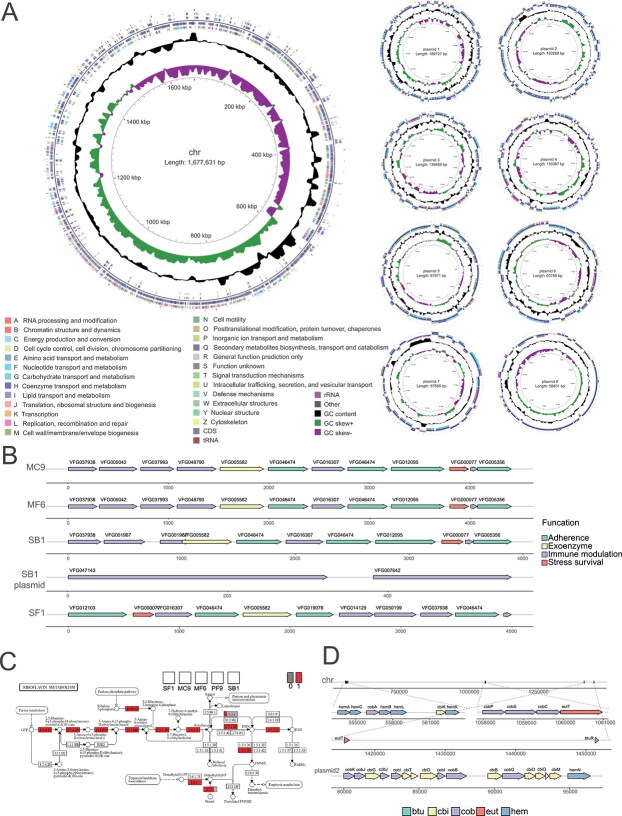
Genomic features and probiotic-related functions of representative *Cetobacterium* strains. (A) Circular genome map of strain SF1 showing genome size, GC content, and GC skew, with coding sequences annotated by eggNOG functional categories. (B) Comparative genomic organization of representative *Cetobacterium* strains highlighting genes associated with probiotic-related functions, including adherence, immune modulation, and stress survival. (C) Reconstruction of the riboflavin/vitamin B_2_–related metabolic pathways based on KEGG annotation, illustrating the completeness of vitamin biosynthesis potential, particularly in strain SF1. (D) Genomic organization of VB_12_ biosynthetic genes.

The complete chromosome of strain SF1 was 1 677 631 bp in length (Fig. 7A). Functional annotation based on the eggNOG database showed a uniform distribution of genes across the genome. These genes are primarily involved in energy production and conversion, amino acid transport and metabolism, carbohydrate transport and metabolism, and coenzyme transport and metabolism. This functional repertoire is consistent with the ecological role of *Cetobacterium* as a metabolically active, facultative anaerobic fermentative bacterium.

To further evaluate the probiotic potential of *Cetobacterium*, functional annotation was conducted with a particular focus on genes associated with beneficial host interactions. Comparative analysis against the Virulence Factor Database revealed that all five *Cetobacterium* isolates harbored genes related to adherence and immune modulation (Fig. 7B). Rather than mediating pathogenicity, these determinants likely facilitate competitive niche exclusion and stable colonization within the fish gut.

All five strains possessed a complete cobalamin biosynthesis pathway (Fig. 7C), with SF1 exhibiting the most comprehensive gene complement for this pathway. Furthermore, gene cluster-based annotation indicated that SF1 harbors all genes required for the de novo biosynthesis of VB_12_ (Fig. 7D). Although the SF1 strain contains the fewest annotated functional genes (924 in total; Fig. S3), it exhibits no deficiencies in core metabolic functions, particularly with regard to vitamin biosynthetic pathways. These genomic signatures suggest a robust metabolic capacity and probiotic potential, though *in vivo* studies are necessary to confirm the physiological efficacy in aquaculture systems.

### *In vitro* assessment of probiotic properties

**Figure 8:**
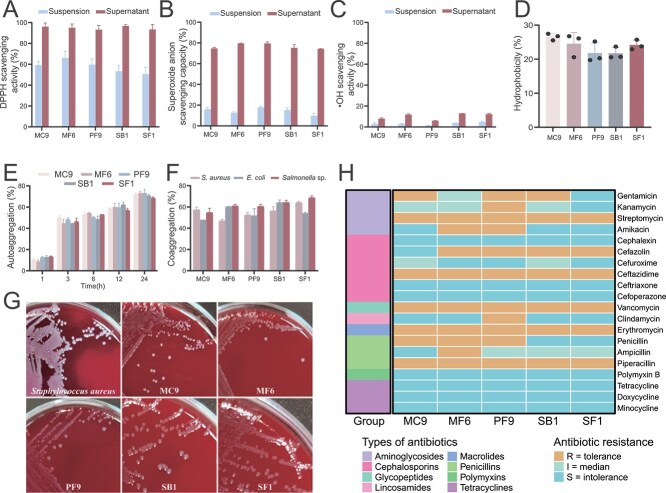
Antioxidant activity and other probiotic properties of representative *Cetobacterium* strains. A–C antioxidant activities of different *Cetobacterium* strains: DPPH scavenging (A), superoxide anion scavenging (B), and hydroxyl radical scavenging (C) activity were measured in both suspension and supernatant. Results indicate that all strains exhibited significant antioxidant activity, with supernatant showing higher activity. (D) Hydrophobicity of bacterial cells from different strains was measured. (E) Autoaggregation ability of the strains at different time points (1, 3, 6, 12, and 24 h). (F) Coaggregation ability with pathogenic strains (*S. aureus*, *E. coli*, *Salmonella*). (G) Hemolytic activity test of the bacterial strain. (H) Antibiotic resistance profiles of different *Cetobacterium* strains.

The *in vitro* antioxidant capacity of both cell-free supernatants (CFS) and intact cells of the five *Cetobacterium* isolates was evaluated using 2,2-diphenyl-1-picrylhydrazyl (DPPH), 2,2'-azino-bis(3-ethylbenzothiazoline-6-sulfonic acid) (ABTS), and hydroxyl radical scavenging assays (Fig. 8A–C). All strains exhibited antioxidant activity, with the CFS was significantly higher than that of the corresponding cellular fractions. This indicates that *Cetobacterium* secretes metabolites possessing antioxidant activity, suggesting a potential role in mitigating intestinal oxidative stress in the host via a secretory mechanism.

All tested strains exhibited moderate-to-low surface hydrophobicity, with indices ranging from 21% to 27% (Fig. 8D), suggesting that adhesive capacity likely depends more on specific molecular interactions than on non-specific hydrophobic adsorption. Correspondingly, auto-aggregation assays revealed that all strains exhibited significant time-dependent aggregation (Fig. 8E), reaching 30%–40% after 4 h and 68%–75% after 24 h. Beyond auto-aggregation, the *Cetobacterium* strains demonstrated a significant ability to co-aggregate with common intestinal pathogens. *In vitro* co-aggregation assays with *Escherichia coli* and *S. aureus* (Fig. 8F) revealed co-aggregation rates ranging from 44% to 71% among the different strains. Regarding safety, no strains produced hemolytic zones on blood agar plates, exhibiting a phenotype consistent with the negative control (Fig. 8G). Antibiotic susceptibility testing revealed that all strains were sensitive to a panel of commonly used clinical antibiotics (Fig. 8H), with no acquired resistance phenotypes detected.

## Discussion

### How diet reshapes the gut microbiome through ecological networks

Dietary changes serve as a primary driver for remodeling the functional and taxonomic architecture of fish gut microbiota [13, 14, 49, 50]. For instance, artificial feeding significantly enhances microbial diversity in *S. chuatsi* compared to live-bait groups. Conversely, microalgae-based diets result in a diversity reduction in *M. salmoides.* These dietary transitions also induce systemic shifts in the relative abundance of dominant phyla in *P. fulvidraco*, thereby dictating the enzymatic capacity of the microbiome, such as cellulase activity in *H. molitrix*, echoing the multi-omic findings observed in *C. idella* [51]. Metagenomic analyses revealed pronounced differences in gut microbial composition among fish with distinct dietary strategies. In carnivorous and carnivory-biased fish (e.g. *M. salmoides* and *S. chuatsi*), members of the genus *Clostridium* within Firmicutes predominated, whereas the gut microbiome of herbivorous fish (e.g. *C. idella*) was dominated by *Aeromonas* within Proteobacteria. These taxonomic rearrangements are intrinsically linked to the reconfiguration of microbial co-occurrence networks [30, 52].

Beyond taxonomic shifts, dietary strategies fundamentally restructure interspecies co-occurrence patterns. Carnivorous fish exhibited higher complexity and lower modularity, reflecting densely interconnected community structures. Such dense networks may promote efficient resource transformation and energy acquisition but are potentially more vulnerable to the loss of keystone taxa. Conversely, herbivorous and filter-feeding fish formed highly modular networks composed of relatively independent functional modules. This modularity enhances community resilience by isolating dietary or environmental perturbations within specific functional modules, thereby preventing systemic collapse [31, 53, 54]. Given that the fish in our study co-occurred within adjacent aquaculture facilities, we speculate that the observed differences in network structure are primarily attributable to interspecific dietary variations. These contrasting network architectures highlight fundamental differences in microbial niche construction, functional coordination, and community stability across dietary strategies [2].

### Functional coordination and redundancy reflect niche specialization

Despite taxonomic and network divergence, the gut microbiomes of the five freshwater fish exhibited highly organized and strategic functional configurations. The conservation of pathways involved in carbohydrate metabolism, amino acid metabolism, energy metabolism, and genetic information processing. This cross-host and cross-diet functional conservation reflects strong functional constraints imposed by the gut environment and suggests that functional redundancy plays a critical role in maintaining the stability of gut microbial ecosystem functions. Furthermore, the functional category “Metabolism of cofactors and vitamins” in *M. salmoides*, *P. fulvidraco*, and *S. chuatsi* suggests host-specific functional investment. In conjunction with the known vitamin-producing capacity of *Cetobacterium*, our findings suggest that *Cetobacterium* serves as a pivotal functional node contributing to vitamin biosynthesis within the gut microbiota of carnivorous fish gut.

Functional convergence observed at broad levels was progressively decoupled at finer resolutions, including KEGG Level 2 pathways, CAZyme families, and ARG categories. At these granular levels, gut microbial functions exhibited strong coordination with host dietary strategies. In particular, the gut microbiome of herbivorous fish was enriched in CAZyme families associated with plant cell wall degradation (e.g. *GH3*, *GH5*, and *GH43*), substantially enhancing the capacity to deconstruct complex plant polysaccharides. This suggests that community-scale functional coordination arises from the strategic recombination of multiple functional modules rather than simple pathway substitution [55].

KEGG Reporter score enrichment analysis revealed that coordination occurred at the level of functional modules. Distinct hosts selectively enriched different functional modules, such as carbohydrate and amino acid-centered modules in herbivorous fish and signaling, nutrient sensing, and environmental-response-related modules in carnivorous or omnivorous fish. This modular enrichment pattern indicates that gut microbial communities adapt to host nutritional strategies by integrating multiple interconnected metabolic and regulatory pathways rather than relying on single “keystone” functions [56]. Functional coordination did not come at the expense of system stability. Core metabolic pathways were supported by multiple phylogenetically distinct microbial taxa, indicating substantial functional redundancy. Such redundancy is thought to buffer fluctuations in community composition and environmental conditions, thereby enhancing the functional resilience of the gut microbiomes [57].

### Freshwater fish intestine as an ecological niche driving evolution and functional specialization of symbiotic microbes

The concurrent emergence of distinct network architectures and functional differentiation indicates that the freshwater fish gut is not merely a passive habitat for microbial colonization. Instead, it represents an active ecological niche that continuously imposes selective pressures, driving the evolution and functional specialization of symbiotic microbes. Our results show that both co-occurrence network topology and functional module enrichment patterns exhibit strong host specificity across different dietary strategies. This concordance suggests that community structure and function are not independently assembled but are instead co-shaped through long-term host–microbe interactions [52].

At the ecological network level, the divergent co-occurrence patterns in carnivorous, herbivorous, and filter-feeding fish represent emergent properties shaped by the host's digestive landscape rather than transient environmental responses. These topologies are not transient responses to short-term environmental fluctuations, but rather emergent properties shaped by the consistent selective filters of the host's digestive landscape. Modular co-occurrence architectures are associated with functional compartmentalization, potentially insulating the microbial community against localized perturbations by limiting the propagation of disturbances. Although the specific arrangement of modules is critical for such stability, highly connected networks may instead facilitate functional coordination and metabolic streamlining, albeit at the risk of increased vulnerability to the loss of key taxa [30].

At the functional level, host-specific enrichment of metabolic pathways and functional modules further supports the view of the gut as a niche for functional specialization. The maintenance of a stable functional core, coupled with the targeted recruitment of accessory modules (e.g. carbohydrate degradation and signal transduction), aligns with the “stable core-plastic periphery” evolutionary framework. This trajectory reflects how symbiotic microbes diversify within resource-stable ecological niches.

Collectively, our findings support that the freshwater fish gut is a dynamic ecological niche that continuously shapes symbiotic microbial community structure and evolutionary trajectories. This process is driven not only by diet-derived resource architecture but also by strain-level heterogeneity and specialized interaction networks [58]. Such niche properties provide the ecological foundation for the functional reinforcement of core symbionts, such as *Cetobacterium* SF1, which exhibits specific genomic adaptations for nutrient acquisition and host colonization. These results support a conceptual framework in which host-associated microbial diversity is maintained through environmental filtering and beneficial interactions, ensuring the functional stability of the core microbiota across diverse aquatic habitats [58].

### Enrichment of *Cetobacterium* in carnivorous fish intestines and its evolutionary trajectory toward genome streamlining

Although detected across all hosts, *Cetobacterium* was predominantly enriched in carnivorous fish (*M. salmoides* and *S. chuatsi*) and carnivore-biased omnivorous fish (*P. fulvidraco*). This distribution pattern suggests that the ecological fitness of *Cetobacterium* may be closely linked to the nutritional landscape of carnivorous gut. Diets dominated by proteins and lipids, with relatively low inputs of complex plant polysaccharides. Such conditions are likely to favor microbial taxa capable of efficiently participating in amino acid metabolism and fermentative processes [59].

VB₁₂ is an essential cofactor for the host but cannot be synthesized de novo by vertebrates. Its availability may be more variable in animal-based dietary systems [60, 61]. Herbivorous fish are generally less constrained by VB₁₂ limitation, likely due to their gut environments being permissive to *in situ* microbial biosynthesis [61]. In this study, all isolated *Cetobacterium* strains harbored complete VB₁₂ biosynthesis pathways. This functional trait may be under positive selection in carnivorous gut environments, thereby facilitating stable colonization and relative enrichment. This reflects an ecological filtering process rather than active host selection, whereby specific nutritional regimes favor strains with essential metabolic contributions.

Comparative genomic analyses revealed that *Cetobacterium* possesses an open pan-genome structure (Fig. 6C), achieving niche adaptation through accessory gene turnover, whereas maintaining a conserved core. *Cetobacterium* SF1 exhibited a markedly reduced genome size (924 genes) compared with the other four *Cetobacterium* strains (>1050 genes), indicating a more streamlined genomic architecture. Despite this reduction, SF1 contained a relatively enriched repertoire of genes associated with VB₁₂ biosynthesis, suggesting that it may adopt a streamlined evolutionary strategy that prioritizes key functional repertoires [62].

Previous studies have proposed two dichotomous microbial adaptive strategies: (i) a streamlined strategy, characterized by genome reduction, high metabolic efficiency, and specialization toward core functions; and (ii) a multifunctional strategy, characterized by larger genomes and broader functional versatility [62, 63]. Streamlined microbiome strategies are typically observed in environments that are relatively stable, nutritionally uniformity, and subject to lower competitive pressures [62]. The carnivorous fish gut, characterized by consistent high-protein inputs and simplified resource spectra, may therefore *Cetobacterium* SF1 facilitate the persistence through “efficient but minimal” genomic configurations for specific dominant taxa. In this niche, specialized lineages may shed genomic redundancy to maximize resource utilization efficiency.

The isolation of *Cetobacterium* strains exclusively from carnivorous and carnivore-biased fish further supports this interpretation. In these hosts, *Cetobacterium* may exist in a more metabolically independent symbiotic state, whereas in herbivorous or filter-feeding fish, its persistence may rely more heavily on complex community interactions and cross-feeding networks. This finding reinforces the fish gut as an evolutionary niche that differentially shapes life-history strategies of core symbionts under distinct dietary contexts.

This integrated analysis of the gut microbiomes of five representative freshwater fish species highlights the gut as a dynamic ecological arena where dietary niches drive significant microbial functional specialization. The open pangenome structure in *Cetobacterium*, coupled with the streamlined genomic repertoire of the carnivorous-specific *Cetobacterium* SF1, provides a compelling case study of lineage-specific adaptation within specialized trophic habitats. By transitioning from taxonomic surveys to niche-based functional and comparative genomics, this study establishes a foundational framework for understanding microbial responses to dietary shifts in aquaculture. Although this study focuses on five representative species, future research incorporating broader host phylogeny and environmental variables will be essential to validate these trophic-driven patterns globally. Ultimately, these insights provide the ecological scaffolding needed to engineer resilient microbiomes for the future of sustainable aquaculture.

## Supplementary Material

FigureS1_wrag125

FigureS2_wrag125

FigureS3_wrag125

TableS1_wrag125

Supplementary_material_for_composting_wrag125

## Data Availability

All data generated or analyzed during this study are included in this published article and its Additional file. All of the original sequences obtained in this work have been deposited in the National Center for Biotechnology Information (NCBI) under project number PRJNA1399069 (https://dataview.ncbi.nlm.nih.gov/object/PRJNA1399069). The relevant code has been uploaded to GitHub (https://github.com/hyShen-hzau/Dietary-Niches-Drive-Microbial-Community-Assembly).
