# Supplementary figures and images for: Dietary niches drive microbial community assembly, network reorganization, and symbiont evolution in freshwater fish gut microbiomes
Source: ISME J. 2026 May 15;20(1):wrag125. doi: 10.1093/ismejo/wrag125 (PMC13249080; doi:10.1093/ismejo/wrag125)

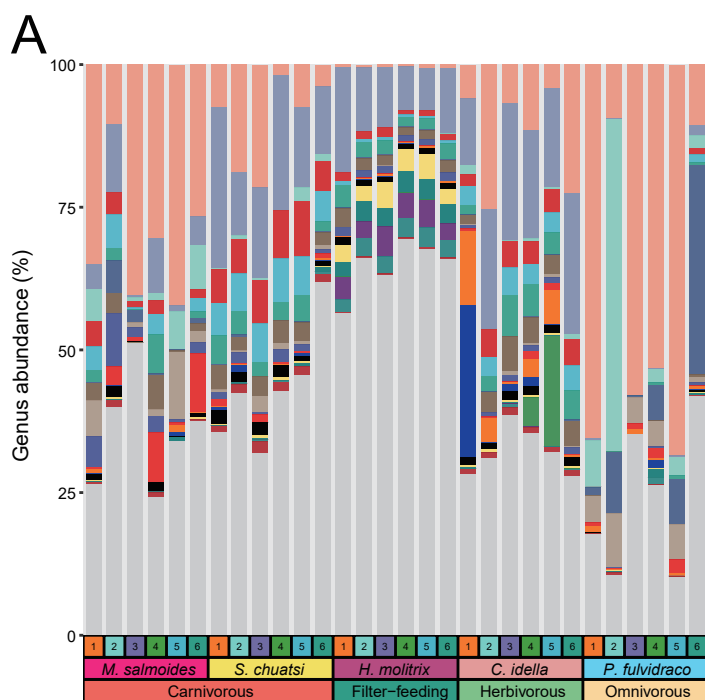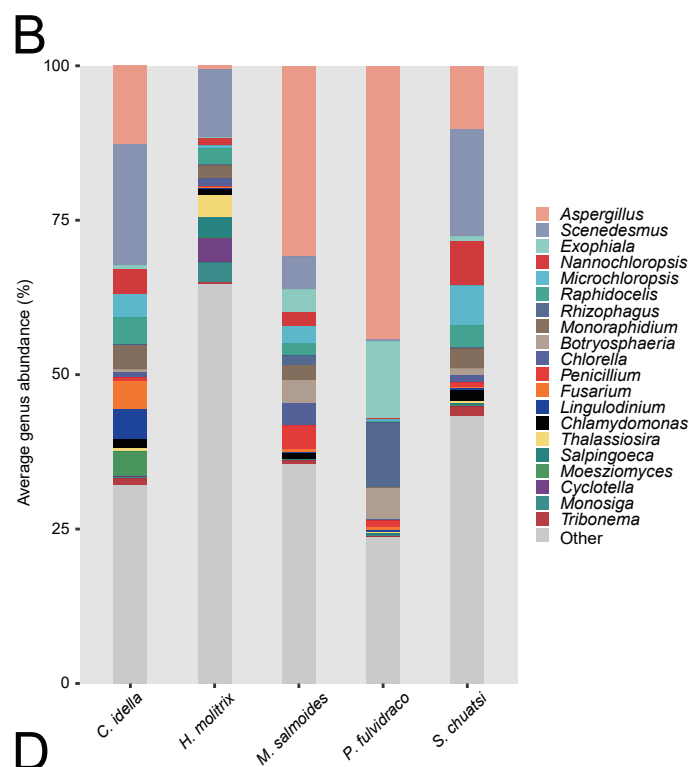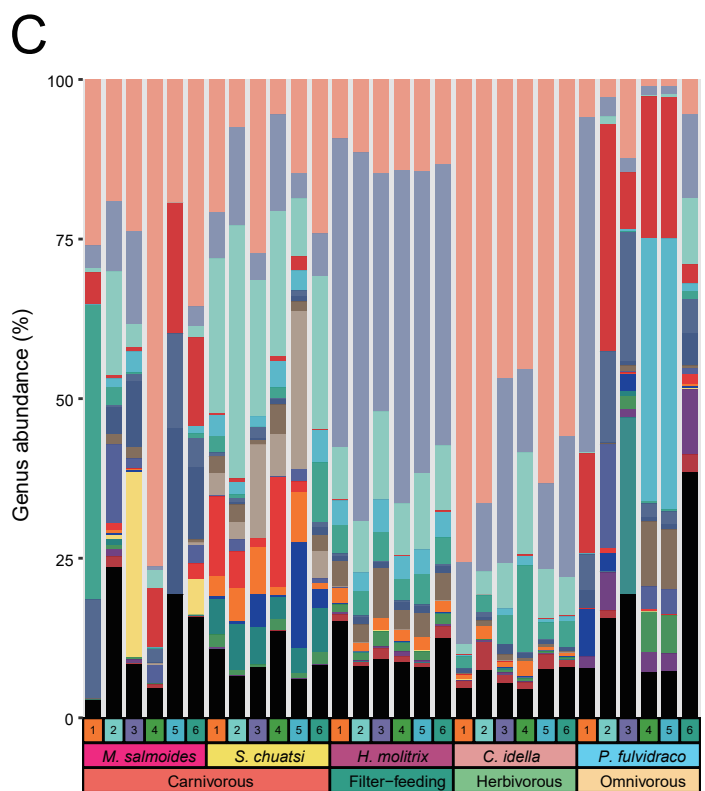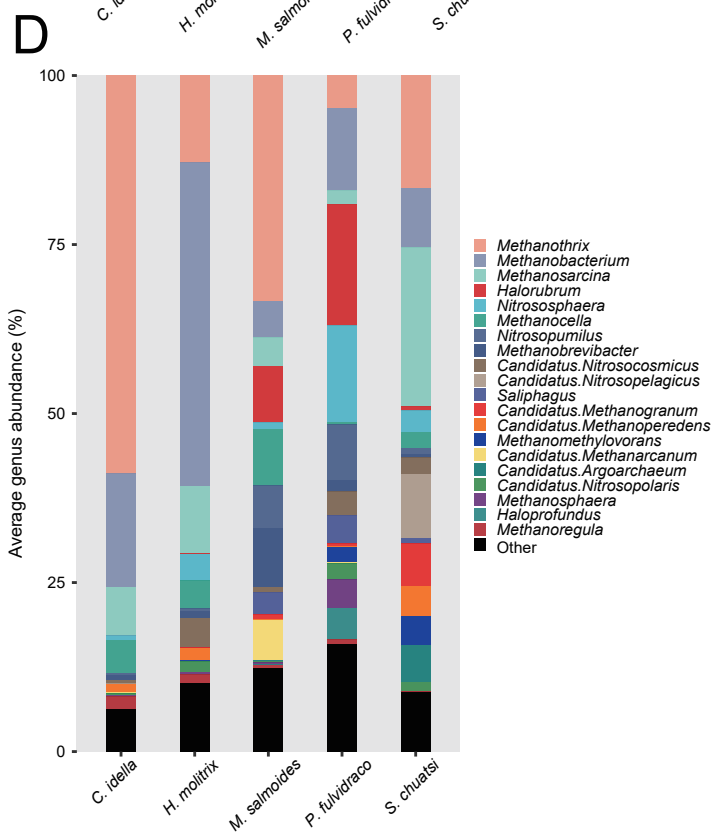

Supplement: FigureS1_wrag125 [file figures1_wrag125.pdf]

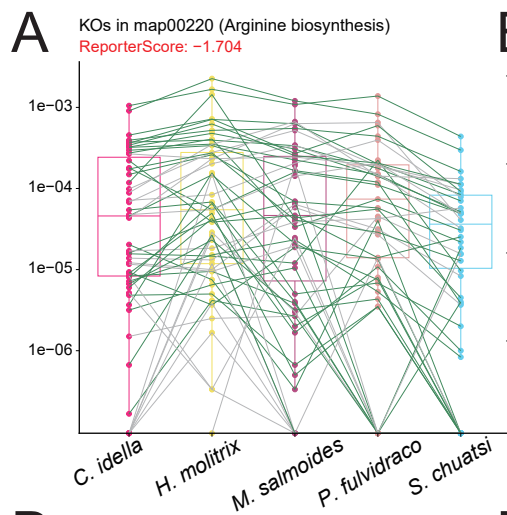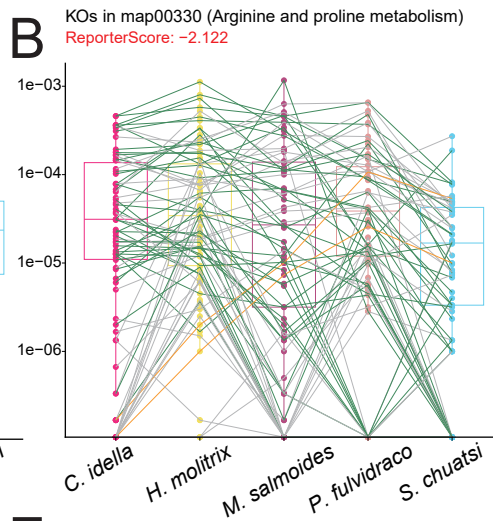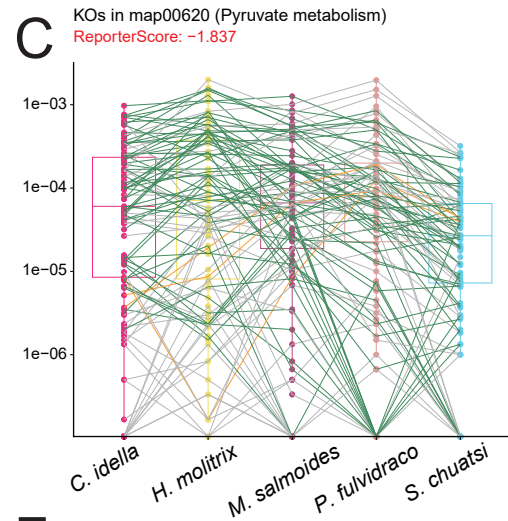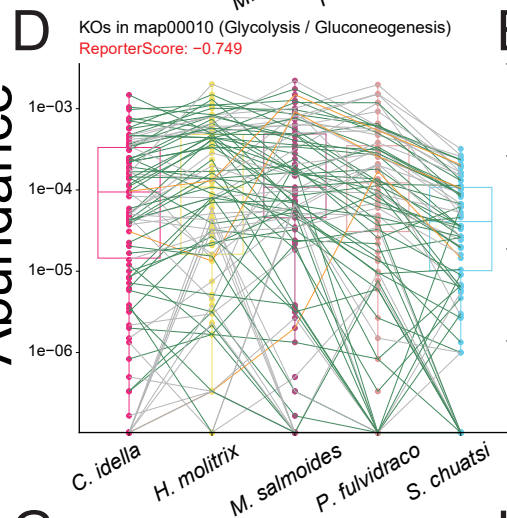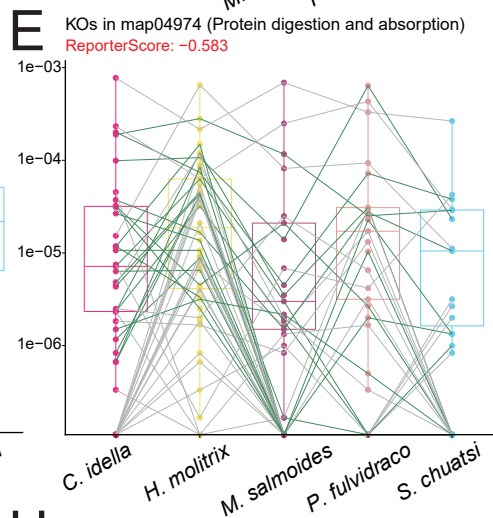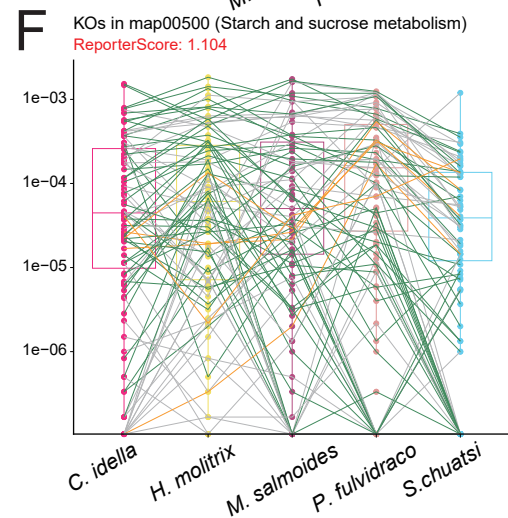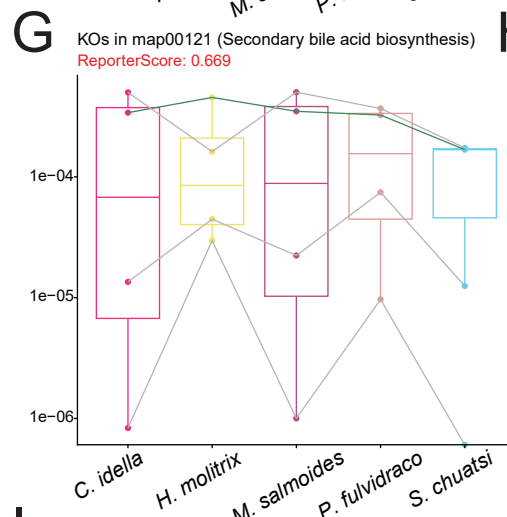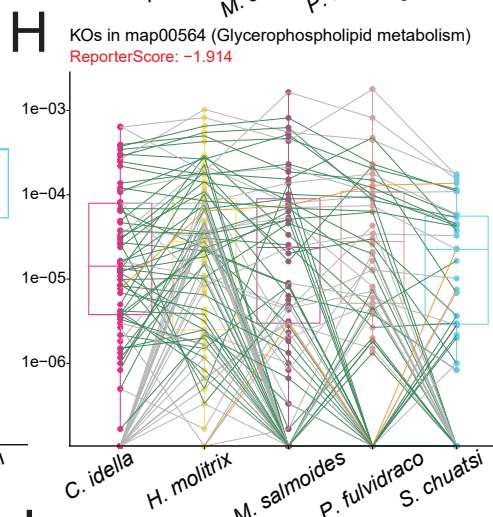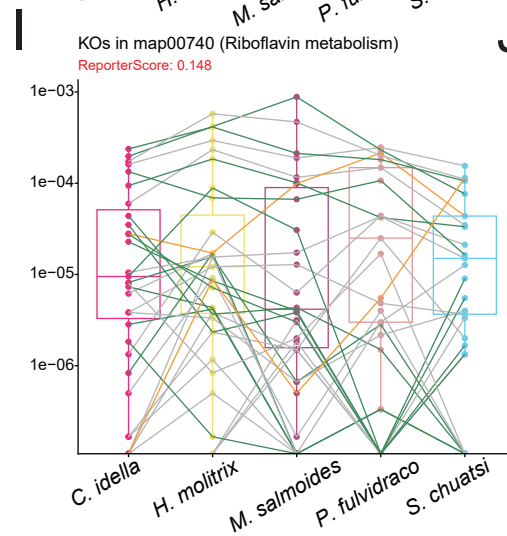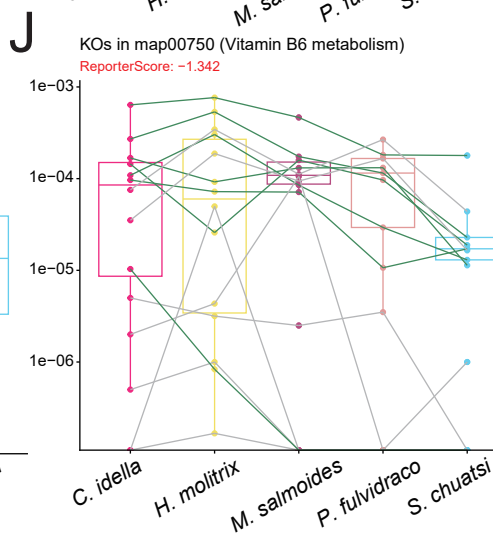

Significantly

— Depleted

— Enriched

— None

Supplement: FigureS2_wrag125 [file figures2_wrag125.pdf]

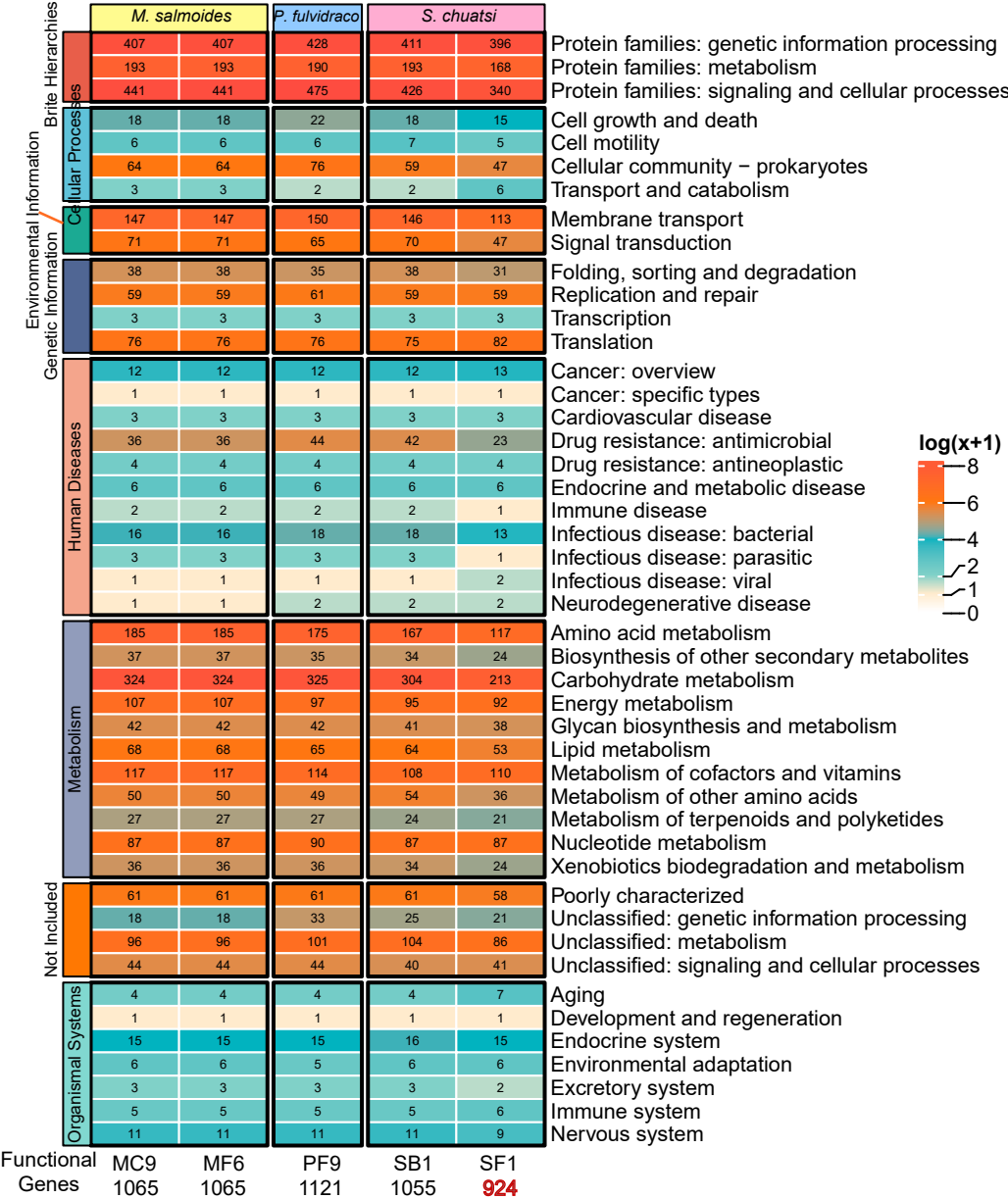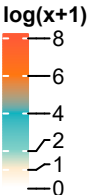

Supplement: FigureS3_wrag125 [file figures3_wrag125.pdf]
