# Supplementary material for: Dietary niches drive microbial community assembly, network reorganization, and symbiont evolution in freshwater fish gut microbiomes
Source: ISME J. 2026 May 15;20(1):wrag125. doi: 10.1093/ismejo/wrag125 (PMC13249080; doi:10.1093/ismejo/wrag125)
Supplement: TableS1_wrag125 [file tables1_wrag125.docx]

Table.S1 Genomic information of 21 *Cetobacterium* strains

| **BioSample ID** | **Strain name** | **Host** | **Completeness** | | **Contamination** | **Isolation Country** | **CollectionDate** |
| --- | --- | --- | --- | --- | --- | --- | --- |
| SAMN15549745 | *Cetobacterium* sp. 2A | *Ictalurus punctatus* | 98.15% | 6.88% | | United States | 2019 |
| SAMN15678418 | *Cetobacterium* sp. 2G large | *Ictalurus punctatus* | 97.12% | 1.38% | | United States | 2019 |
| SAMN15678419 | *Cetobacterium* sp. 8H | *Ictalurus punctatus* | 99.03% | 1.04% | | United States | 2019 |
| SAMN02745174 | *Cetobacterium* *ceti* ATCC 700028 | *Balaenoptera acutorostrata* | 98.77% | 0.94% | | United Kingdom | 2014 |
| SAMN44522929 | *Cetobacterium* *someae* ATCC BAA-474 | *Homo sapiens* | 96.94% | 1.46% | | United States | 2024 |
| SAMN37189466 | *Cetobacterium* *colombiensis* C33 | *Oreochromis niloticus* | 98.67% | 2.12% | | Colombia | 2018 |
| SAMN39433506 | *Cetobacterium* *someae* ceto | *Pelteobagrus fulvidraco* | 99.19% | 1.82% | | China | 2023 |
| SAMN26150816 | *Cetobacterium* *someae* CS2105-BJ | *Danio rerio* | 99.12% | 1.52% | | China | 2021 |
| SAMN31676781 | *Cetobacterium* *someae* LJ | *Micropterus salmoides* | 98.32% | 0.94% | | China | 2021 |
| SAMN34079186 | *Cetobacterium* *someae* MSU41 | *Ictalurus punctatus × I. furcatus* | 88.95% | 0.78% | | United States | 2022 |
| SAMN34079187 | *Cetobacterium* *someae* MSU49 | *Ictalurus punctatus × I. furcatus* | 90.63% | 1.04% | | United States | 2022 |
| SAMN29837394 | *Cetobacterium* sp. NK01 | *Oreochromis niloticus* | 99.03% | 1.39% | | China | 2021 |
| SAMN15095610 | *Cetobacterium* *someae* zfcc0105 | *Danio rerio* | 99.06% | 2.12% | | United States | 2020 |
| SAMN48716859 | *Cetobacterium* *someae* ZNN-1 | *Oreochromis niloticus* | 98.67% | 0.79% | | China | 2022 |
| SAMN03021532 | *Cetobacterium* sp. ZOR0034 | *Danio rerio* | 99.29% | 0.60% | | United States | 2012 |
| SAMN03021539 | *Cetobacterium* sp. ZWU0022 | *Danio rerio* | 99.29% | 0.41% | | United States | 2012 |
| SAMN54440622 | *Cetobacterium* *someae* MC9 | *Micropterus salmoides* | 99.29% | 1.73% | | China | 2024 |
| SAMN54440671 | *Cetobacterium* *someae* MF6 | *Micropterus salmoides* | 99.29% | 1.73% | | China | 2024 |
| SAMN54440731 | *Cetobacterium* *someae* PF9 | *Pelteobagrus fulvidraco* | 99.19% | 0.94% | | China | 2024 |
| SAMN54440760 | *Cetobacterium* *someae* SB1 | *Siniperca chuatsi* | 99.12% | 1.73% | | China | 2024 |
| SAMN48112985 | *Cetobacterium* *ceti* SF1 | *Siniperca chuatsi* | 99.29% | 1.32% | | China | 2024 |
