# Supplementary material for: Dietary niches drive microbial community assembly, network reorganization, and symbiont evolution in freshwater fish gut microbiomes
Source: ISME J. 2026 May 15;20(1):wrag125. doi: 10.1093/ismejo/wrag125 (PMC13249080; doi:10.1093/ismejo/wrag125)
Supplement: Supplementary_material_for_composting_wrag125 [file supplementary_material_for_composting_wrag125.docx]

**Supplementary material for**

**Dietary Niches Drive Microbial Community Assembly, Network Reorganization, and Symbiont Evolution in Freshwater Fish Gut Microbiomes**

Hongye Shen^1^, Jinhui Song^1^, Jinshan Li^1^, Yongmei Hu^1^, Nan Peng^1^, Shumiao Zhao^1^*****

^1^ National Key Laboratory of Agricultural Microbiology and College of Life Science and Technology, Huazhong Agricultural University, Wuhan 430070, China

**This supplementary file includes:**

**Appendix for materials and methods:**

Appendix 1 Method for DNA extraction and MiSeq sequencing

Appendix 2 Determination of Antioxidant Capacity

Appendix 3 Hemolytic Activity Assay

Appendix 4 Microbial co-occurrence networks construction and characterization

Appendix 5 Comparative Genomics Analysis of *Cetobacterium*

**2. Supplementary figures and tables**

Fig. S1: Gut microbiome profiles of five freshwater fish species with distinct feeding habits (Eukaryotes and Archaea) (A) Genus-level eukaryotic community composition across individual samples. (B) Average relative abundance of dominant eukaryotic genera in each fish species. (C) Genus-level archaeal community composition across individual samples. (D) Average relative abundance of dominant archaeal genera in each fish species.

Fig. S2: Differential results regarding the functional enrichment of specific pathways in the gut microbiomes of different fish species.

Fig. S3: Heatmap of the functional gene composition of the five *Cetobacterium* strains.

Table.S1 Genomic information of 21 *Cetobacterium* strains

**Appendix 1 Method for DNA extraction and Metagenomic sequencing**

**DNA extraction, Library preparation and Sequencing**

Total microbial genomic DNA was extracted using the MagBeads FastDNA Kit for Soil (Catalog No. 116564384; MP Biomedicals, CA, USA) according to the instructions of the manufacturer. The DNA samples were stored at −20°C prior to further analysis. The quantity of the extracted DNA was determined using a Qubit 4 Fluorometer (Invitrogen, Carlsbad, CA, USA) with the Qubit 1X dsDNA HS Assay Kit (Catalog No. Q33231), and the DNA quality and integrity were assessed via 1% (w/v) agarose gel electrophoresis.

Metagenomic shotgun sequencing libraries were constructed with a target insert size of 400 bp using the Illumina TruSeq Nano DNA LT Library Preparation Kit (Illumina, San Diego, CA, USA). Sequencing was performed on the Illumina NovaSeq 6000 platform (Illumina) using a paired-end 150 bp (PE150) strategy.

**Appendix 2 Determination of Antioxidant Capacity**

**2.1 Preparation of Bacterial Cell Suspensions**

The activated *Cetobacterium* strains were inoculated into liquid Gifu Anaerobic Medium (GAM) at a 1% (v/v) ratio and incubated at 37°C for 24 h under anoxic conditions. To separate the bacterial fractions, the cultures were centrifuged at 4,000 × g for 10 min at 4°C. The resulting supernatant was collected and passed through a 0.22 μm pore-size polyethersulfone membrane filter to obtain the cell-free supernatant (CFS), which was stored at 4°C for subsequent assays. The remaining bacterial pellets were washed twice with ice-cold sterile phosphate-buffered saline (PBS) and resuspended in an equal volume of PBS to obtain the intact cell suspension. All preparation steps were performed under stringent environmental controls to maintain cell viability and metabolic integrity.

**2.2 Scavenging Activity of DPPH Radical**

The 2,2-diphenyl-1-picrylhydrazyl (DPPH) radical scavenging capacity was determined as previously described with minor modifications. Briefly, 1 mL of cell-free supernatant or cell suspension was mixed with 1 mL of DPPH solution (0.2 mmol/L in absolute ethanol). The mixture was shaken and incubated in the dark at room temperature for 30 min. After centrifugation at 6,000 × g for 10 min at 4°C, the absorbance of the supernatant was measured at 517 nm (A_i_). For the blank group (A_j_), absolute ethanol was used instead of the DPPH solution, and for the control group (A_0_), distilled water was used instead of the sample. A mixture of distilled water and absolute ethanol was used to zero the spectrophotometer. The DPPH scavenging rate was calculated as follows:

DPPH scavenging rate (%) = [1-(Ai-Aj)/A_0_] × 100%

**2.3 Inhibition of Hydroxyl Radical**

The hydroxyl radical (-OH) inhibition capacity was measured using a commercial hydroxyl radical assay kit (Nanjing Jiancheng Bioengineering Institute, China) based on the Fenton reaction principle. The absorbance was measured at 550 nm, and the inhibition rate was calculated according to the instructions of the manufacturer.

**2.4 Superoxide Anion Scavenging Activity**

Superoxide anion scavenging capacity was evaluated using the pyrogallol autoxidation method. A reaction mixture was prepared by adding 0.5 mL of cell-free supernatant to a solution containing 1 mL each of Tris-HCl (150 mmol/L, pH 8.2), pyrogallol (1.2 mmol/L), and diethylenetriaminepentaacetic acid (3 mmol/L). The final volume was adjusted to 3.5 mL. Following incubation in a water bath at 25°C for 10 min, the absorbance was measured at 325 nm. The scavenging rate was calculated as:

Superoxide anion scavenging rate (%) = [1 - (A_11_ - A_10_) / (A_01_ - A_00_)] ×100%

- A_00_: Absorbance without sample and pyrogallol.
- A_01_: Absorbance without sample but with pyrogallol.
- A_10_: Absorbance with sample but without pyrogallol.
- A_11_: Absorbance with both sample and pyrogallol.

**Appendix 3** **Hemolytic Activity Assay**

The hemolytic activity of *Cetobacterium* isolates was evaluated using Columbia blood agar supplemented with 5% (v/v) sterile sheep blood (Jiangsu ZhengSheng Biotechnology Co., Ltd., China). Bacterial cell suspensions were standardized to a concentration of 10^8^ CFU/mL prior to inoculation. Aliquots (10 μL) of the standardized suspension were inoculated onto the blood agar plates and incubated at 30°C for 48 h, pH 7.2. To simulate the intestinal environment of the freshwater fish hosts, incubation was performed under a strictly anaerobic atmosphere (80% N_2_, 10% CO_2_, 10% H_2_).

Hemolysis was assessed by observing the formation of clear zones (β-hemolysis), greenish zones (α-hemolysis), or no zones (γ-hemolysis) around the colonies.

**Appendix 4 Microbial co-occurrence networks construction and characterization**

We constructed a metacommunity cross-kingdom co-occurrence network of all samples and extracted subnetworks.

Microbial co-occurrence networks were constructed and visualized using the R package “ggNetView” (available at https://github.com/Jiawang1209/ggNetView). To avoid potential spurious associations of rare OTUs from affecting reliability, data filtering was performed prior to correlation calculation. For the metacommunity ross-kingdom co-occurrence network, only OTUs present in 6 of the 30 samples were included for calculation. To minimize noise within the networks, a stringent Spearman correlation threshold (r > 0.8) and significance level (*P* < 0.01) were applied, with p values further adjusted using the Benjamini-Hochberg (BH) method.

A metacommunity co-occurrence network was initially established based on Pearson correlation and the Random Matrix Theory (RMT)-based approach. Subsequently, sample-specific sub-networks were extracted using the induced subgraph function in the “igraph” package. We quantified the topological properties of each sub-network—including the number of nodes and edges, average degree, clustering coefficient, and betweenness centrality—as indicators of network complexity. To evaluate network stability, we calculated robustness (defined as the proportion of remaining nodes after simulated random node loss) and vulnerability (identifying nodes whose removal significantly impacts network efficiency). These metrics were used to compare the organizational stability of gut microbial communities across different dietary niches.

Comprehensive details regarding network construction and characterization are provided in Supporting Information: Material S1 (Appendix 4).

**Appendix 5** **Comparative Genomics Analysis of *Cetobacterium***

**5.1 Genome Collinearity Analysis**

To evaluate genomic structural variations, whole-genome collinearity analysis was performed using Mauve software (v2.4.0) with the progressive Mauve algorithm. The dataset comprised the chromosomal and plasmid sequences of five isolates obtained in this study (*Cetobacterium* sp. MC9, MF6, PF9, SB1, and SF1) and two type strains (*Cetobacteriu. ceti* ATCC 700028 and *Cetobacterium* *somerae* ATCC BAA-474). All genomic sequences were concatenated into a multi-FASTA file and imported into the software with default parameters. Structural variation events, including the arrangement, inversion, and rearrangement of Locally Collinear Blocks (LCBs), were visualized and analyzed to infer evolutionary relationships among the strains.

**5.2 Pangenome Analysis**

To investigate the genetic diversity and evolutionary trajectories within the genus *Cetobacterium*, a pangenome analysis was conducted using 21 representative strains. This collection included five newly sequenced isolates (MC9, MF6, PF9, SB1, and SF1) and 16 publicly available genomes retrieved from the NCBI database (detailed metadata provided in Table S1).

The pangenome workflow was executed using the Bacterial Pan Genome Analysis (BPGA) pipeline. Protein-coding sequences from all strains were used as input. Orthologous gene clusters (OGCs) were identified using the USEARCH algorithm with a default sequence identity threshold of 50%. The pangenome was categorized into three components:

1. Core Genome: Gene clusters shared across all 21 strains.

2. Accessory Genome: Gene clusters present in two or more strains but not all.

3. Unique Genes: Gene clusters restricted to a single strain.

The openness of the *Cetobacterium* pangenome was assessed using the power-law regression model built into BPGA, fitting the cumulative number of pangenome and core genome clusters against the number of added genomes.

**5.3 Core-genome Phylogenetic Analysis**

To clarify the phylogenetic relationships among the 21 *Cetobacterium* strains and determine the taxonomic position of the potential novel species, a core-genome phylogenetic tree was reconstructed using the Integrated Prokaryotic Genome Analysis (IPGA) platform (https://nmdc.cn/ipga/).

The analysis included 21 high-quality *Cetobacterium* genomes (including the five isolates from this study) and *Escherichia coli* ATCC 11775 as the outgroup. Protein-coding sequences were predicted using Prodigal (v2.6.3), and single-copy core orthologous genes shared across all 22 genomes were identified using OrthoFinder (v2.5.4). Multiple sequence alignments of the identified core genes were performed using MAFFT (v7.490), followed by automated trimming and concatenation to generate a super-matrix of core gene sequences.

The maximum-likelihood (ML) phylogenetic tree was constructed using IQ-TREE (v2.2.0), with the optimal nucleotide substitution model automatically selected by the built-in ModelFinder. Branch support was evaluated using 1,000 ultrafast bootstrap replicates. The resulting Newick tree file was visualized and refined using the TVBOT online tool (https://www.chiplot.online/tvbot.html). The tree was manually rooted using *E. coli* ATCC 11775 as the outgroup, and bootstrap values were annotated at each node to indicate topological reliability.

Table.S1 Genomic information of 21 *Cetobacterium* strains

| **BioSample ID** | **Strain name** | **Host** | **Completeness** | | **Contamination** | **Isolation Country** | **CollectionDate** |
| --- | --- | --- | --- | --- | --- | --- | --- |
| SAMN15549745 | *Cetobacterium* sp. 2A | *Ictalurus punctatus* | 98.15% | 6.88% | | United States | 2019 |
| SAMN15678418 | *Cetobacterium* sp. 2G large | *Ictalurus punctatus* | 97.12% | 1.38% | | United States | 2019 |
| SAMN15678419 | *Cetobacterium* sp. 8H | *Ictalurus punctatus* | 99.03% | 1.04% | | United States | 2019 |
| SAMN02745174 | *Cetobacterium* *ceti* ATCC 700028 | *Balaenoptera acutorostrata* | 98.77% | 0.94% | | United Kingdom | 2014 |
| SAMN44522929 | *Cetobacterium* *someae* ATCC BAA-474 | *Homo sapiens* | 96.94% | 1.46% | | United States | 2024 |
| SAMN37189466 | *Cetobacterium* *colombiensis* C33 | *Oreochromis niloticus* | 98.67% | 2.12% | | Colombia | 2018 |
| SAMN39433506 | *Cetobacterium* *someae* ceto | *Pelteobagrus fulvidraco* | 99.19% | 1.82% | | China | 2023 |
| SAMN26150816 | *Cetobacterium* *someae* CS2105-BJ | *Danio rerio* | 99.12% | 1.52% | | China | 2021 |
| SAMN31676781 | *Cetobacterium* *someae* LJ | *Micropterus salmoides* | 98.32% | 0.94% | | China | 2021 |
| SAMN34079186 | *Cetobacterium* *someae* MSU41 | *Ictalurus punctatus × I. furcatus* | 88.95% | 0.78% | | United States | 2022 |
| SAMN34079187 | *Cetobacterium* *someae* MSU49 | *Ictalurus punctatus × I. furcatus* | 90.63% | 1.04% | | United States | 2022 |
| SAMN29837394 | *Cetobacterium* sp. NK01 | *Oreochromis niloticus* | 99.03% | 1.39% | | China | 2021 |
| SAMN15095610 | *Cetobacterium* *someae* zfcc0105 | *Danio rerio* | 99.06% | 2.12% | | United States | 2020 |
| SAMN48716859 | *Cetobacterium* *someae* ZNN-1 | *Oreochromis niloticus* | 98.67% | 0.79% | | China | 2022 |
| SAMN03021532 | *Cetobacterium* sp. ZOR0034 | *Danio rerio* | 99.29% | 0.60% | | United States | 2012 |
| SAMN03021539 | *Cetobacterium* sp. ZWU0022 | *Danio rerio* | 99.29% | 0.41% | | United States | 2012 |
| SAMN54440622 | *Cetobacterium* *someae* MC9 | *Micropterus salmoides* | 99.29% | 1.73% | | China | 2024 |
| SAMN54440671 | *Cetobacterium* *someae* MF6 | *Micropterus salmoides* | 99.29% | 1.73% | | China | 2024 |
| SAMN54440731 | *Cetobacterium* *someae* PF9 | *Pelteobagrus fulvidraco* | 99.19% | 0.94% | | China | 2024 |
| SAMN54440760 | *Cetobacterium* *someae* SB1 | *Siniperca chuatsi* | 99.12% | 1.73% | | China | 2024 |
| SAMN48112985 | *Cetobacterium* *ceti* SF1 | *Siniperca chuatsi* | 99.29% | 1.32% | | China | 2024 |
